# Consistent individual differences in foraging movement tactics drive habitat partitioning in an inland-breeding Great Cormorant population

**DOI:** 10.1186/s40462-026-00682-6

**Published:** 2026-07-21

**Authors:** Bálint Preiszner, Péter Szinai, Gyula Kovács

**Affiliations:** 1 HUN-REN Balaton Limnological Research Institute, Hungarian Research Network, Klebelsberg K. u. 3., Tihany, H-8237 Hungary; 2 National Laboratory for Water Science and Water Security, HUN-REN Balaton Limnological Research Institute, Klebelsberg K. u. 3., Tihany, H-8237 Hungary; 3 Balaton-felvidéki National Park Directorate, Kossuth u. 16., Csopak, H-8229 Hungary; 4 Bird Ringing and Migration Study Group of BirdLife Hungary, Költő u. 21., Budapest, H-1121 Hungary; 5 BirdLife Hungary South-Balaton Local Group, Irmapuszta, Balatonlelle, H-8638 Hungary

**Keywords:** Individual specialisation, Foraging habitat selection, Freshwater habitat use, GPS tracking, Great Cormorant, Habitat repeatability, Fishpond, Intraspecific niche partitioning, Wildlife-fishery conflict

## Abstract

**Background:**

Individual variation in animal movement and habitat selection is increasingly recognised as a structurally important component of population ecology, yet the magnitude and temporal consistency of foraging movement specialisation remain poorly characterised in inland-breeding waterbirds. Inland populations of Great Cormorants (*Phalacrocorax carbo sinensis*) exploit a mosaic of freshwater habitat types in anthropogenic landscapes and generate recurrent conflict with fisheries. Whether individuals consistently differ in foraging movement tactics across habitat types and seasons has not been established.

**Methods:**

We deployed solar-powered GPS transmitters on 24 Great Cormorants (5 adults, 19 fledglings) from an inland-breeding colony at Lake Balaton, Central Europe, between 2021 and 2023 (mean deployment duration: 190 days). Across six freshwater habitat types (fishponds, rivers, lakes, wetlands, regulated water bodies, river arms/oxbow lakes), we recorded 249,097 daytime fixes and 20,678 speed-classified foraging locations. Individual variation in seasonal foraging habitat use profiles was quantified using PERMANOVA and ANOSIM on Bray–Curtis dissimilarities; temporal consistency was estimated as adjusted repeatability via linear mixed models.

**Results:**

Individual identity accounted for 73.6% of total variation in seasonal habitat use profiles (PERMANOVA, *p* < 0.0001), and between-individual dissimilarities significantly exceeded within-individual dissimilarities (ANOSIM *R* = 0.511, *p* < 0.0001). Age class explained a negligible fraction of variation (R^2^ = 0.012, *p* = 0.887). Principal coordinates analysis revealed a structured partition of individual movement space along three largely independent axes – fishpond, river and lake use (r^2^ = 0.957, 0.949, 0.803; all *p* < 0.001) – jointly accounting for 74.8% of total individual-level variation. Habitat use was significantly repeatable across seasons for four of six habitat types: river (*R* = 0.722), fishpond (*R* = 0.642), regulated water body (*R* = 0.592) and lake (*R* = 0.575; all *p* < 0.001).

**Conclusions:**

Inland-breeding Great Cormorants show strong, temporally stable individual differences in foraging movement tactics across seasons, with distinct subsets of individuals consistently targeting fishponds, rivers or lakes – each to the near-exclusion of the others. Habitat partitioning in this population emerges from consistent individual movement decisions, not ontogenetic niche shifts or opportunistic habitat switching. Population-level management will produce disproportionate, habitat-sector-specific outcomes; habitat-explicit, individually targeted approaches are likely to prove more effective in mitigating cormorant–fishery conflicts in inland landscapes.

**Supplementary information:**

The online version contains supplementary material available at https://doi.org/10.1186/s40462-026-00682-6.

## Background

Great Cormorant (*Phalacrocorax carbo*) is a widespread, colonial piscivore species that exploits a broad spectrum of habitats and breeds in dense, often large colonies throughout its Palearctic range. Great Cormorant conflicts with fisheries are further compounded by a long-standing cultural antagonism toward the species, rooted in its appearance and historical association with ill omen [[1] 2026] – a perception that can complicate evidence-based management. Especially in anthropogenic freshwater landscapes, such as reservoirs, altered riverscapes, and notably fishponds, substantial predation pressure by cormorants on fish stocks is often presumed. This generates recurrent conflicts with commercial and recreational fisheries. Recently even conservation concerns were raised for native fish communities [see eg. [2]]. Therefore, understanding how cormorants move between and select among available habitats is critical for assessing their ecological role and for designing sustainable management strategies that stakeholders of both fisheries and conservation may accept [3, 4]. Recent advances in telemetry technologies made available the detection of fine-scale movement and space use patterns, enabling a better understanding of distributions between habitats. Risk mapping based on such information may directly inform management planning resulting in better assessments [3–5].

*Ph. c. sinensis* is the predominantly inland-breeding subspecies across continental Europe, with subspecies-level differences in habitat preferences documented since early taxonomic work [6], yet fine-scale movement ecology of inland *sinensis* populations remains poorly characterised. Despite the technological progress, knowledge of Great Cormorant movement ecology in certain regions and habitats is still lacking, and significant gaps persist for inland-breeding populations [7, 8]. Early work on cormorant movement was based on ringing and resighting data, which provided dispersal patterns but lacked fine scale temporal and spatial information about daily foraging routes and habitat use. More recent telemetry made fine-scale spatial data available with large amount of data of focal individuals, but such studies have focused mainly on marine or estuarine contexts and have often studied only a few birds that limits among-individual variation analysis [7, 9]. GPS-based tracking of breeding Great Cormorants revealed sex-specific differences in foraging habitat choice and trip characteristics, with some individuals specializing on offshore marine habitats while others feeding exclusively in freshwater. These studies however do not quantify the variation in movement tactics and its potential implications for fisheries in inland populations [7]. In a broader taxonomic context, although general evidence indicates that individual specialization and temporal partitioning can provide basis for coexistence among sympatric cormorant species, the extent to which such individual-level differences shape habitat use in inland-breeding populations remains poorly understood [2].

Specialization in individual foraging tactics may be driven by intraspecific competition, predictability of the habitat, and behavioural plasticity of the individuals. Such specialization may be more expressed in anthropogenic landscapes that provide prey resources structured in space and time [10, 11]. Furthermore, specialization in foraging behaviour may generate further complexity in predator-prey interactions that may have direct management implications in species exploiting resources also used by humans. Telemetry studies documented strong individual consistency in foraging area use across multiple cormorant species, with each bird exploiting a specific subset of habitats within the population’s range [12, 13]. In Great Cormorants, individuals from the same colony can consistently use distinct habitat types; some foraging exclusively in marine waters, some solely in freshwater, while others using both; this suggests persistent habitat preferences rather than opportunistic use of all available areas [7, 14]. Stable isotope analysis in closely related shags confirms such individual specialization in the long-term habitat use, furthered with sex-specific niche partitioning across breeding seasons despite population-level overlap in resource use [15]. In other cormorant species telemetry confirms high foraging-site fidelity during the chick-rearing period, with individuals repeatedly using specific locations for weeks [16, 17]. GPS tracking at a coastal colony of Great Cormorants revealed sex-specific habitat segregation; males avoided freshwater habitats entirely and females exploited marine, estuarine, and inland sites. This pattern is potentially driven by intraspecific competition at nearshore foraging locations [7]. Diet-based evidence from an inland-breeding population likewise revealed sex-specific prey partitioning, with males taking larger and more diverse fish, indicating that intersexual resource differentiation is not confined to coastal systems [18]. Such individual-level partitioning of habitats may have direct implications for management: If certain individuals or demographic groups consistently exploit specific habitat types – for example, fishponds versus natural lakes – then lethal or non-lethal interventions targeting one habitat will fail to mitigate impacts in the other habitat, analogous to the ‘problem individual’ paradigm in seals and other predators, where targeting high-impact specialists yields disproportionate benefits [3, 19]. Therefore, the degree to which inland-breeding Great Cormorants exhibit such individual-level habitat specialization, and whether it persists across seasons, determines the spatial scale and targeting required for effective, evidence-based management actions.

High-resolution movement data from other aquatic predators have revealed habitat use patterns and quantified overlap with human activities, enabling targeted management actions [3–5]. For pinnipeds, large GPS datasets were used to model regional habitat preferences and test whether protected areas cover key marine habitats for them [3]. Fine-scale tracking of diving birds in tidal waters also demonstrated individual selectivity for current speed and turbulence – factors influencing collision risk with marine renewable energy structures [9]. Such work shows that measuring movement diversity and individual habitat choices may help to identify conflict hotspots, but to our knowledge no telemetry studies addressing such issues exist for inland-breeding cormorants. Studying GPS tracking data from inland populations’ habitat use are therefore needed to quantify individual space-use variation across freshwater types and link it to management needs.

We addressed these gaps using high-resolution GPS tracking of an inland-breeding population of *Ph. c. sinensis* at Lake Balaton, Central Europe. Based on the framework of Araújo et al. [10], we tested two complementary hypotheses. First, we predicted that individual identity is the dominant predictor of foraging habitat use, reflecting stable, individually acquired habitat use patterns in an anthropogenic freshwater landscape where fishponds, rivers and large lakes differ markedly in prey predictability and accessibility. Specifically, we expected that (i) between-individual dissimilarity in seasonal habitat use profiles would substantially exceed within-individual dissimilarity, and (ii) habitat use would be repeatable across seasons, while age class would account for a negligible fraction of total variation. Second, we predicted that individual specialisation is non-uniformly distributed across the population, with distinct subsets of individuals consistently exploiting fishponds, rivers or lakes as their primary foraging habitat. Under this hypothesis, (iii) individual variation in habitat use would show a structured pattern in multivariate space, and (iv) individuals sharing a primary foraging habitat would show greater spatial overlap in their foraging networks than individuals using different habitat types. If supported, these patterns would imply that a subset of the population generates disproportionate, habitat-specific predation pressure – justifying a shift from population-level management toward habitat-explicit conflict mitigation.

## Methods

### Study population

**Fig. 1:**
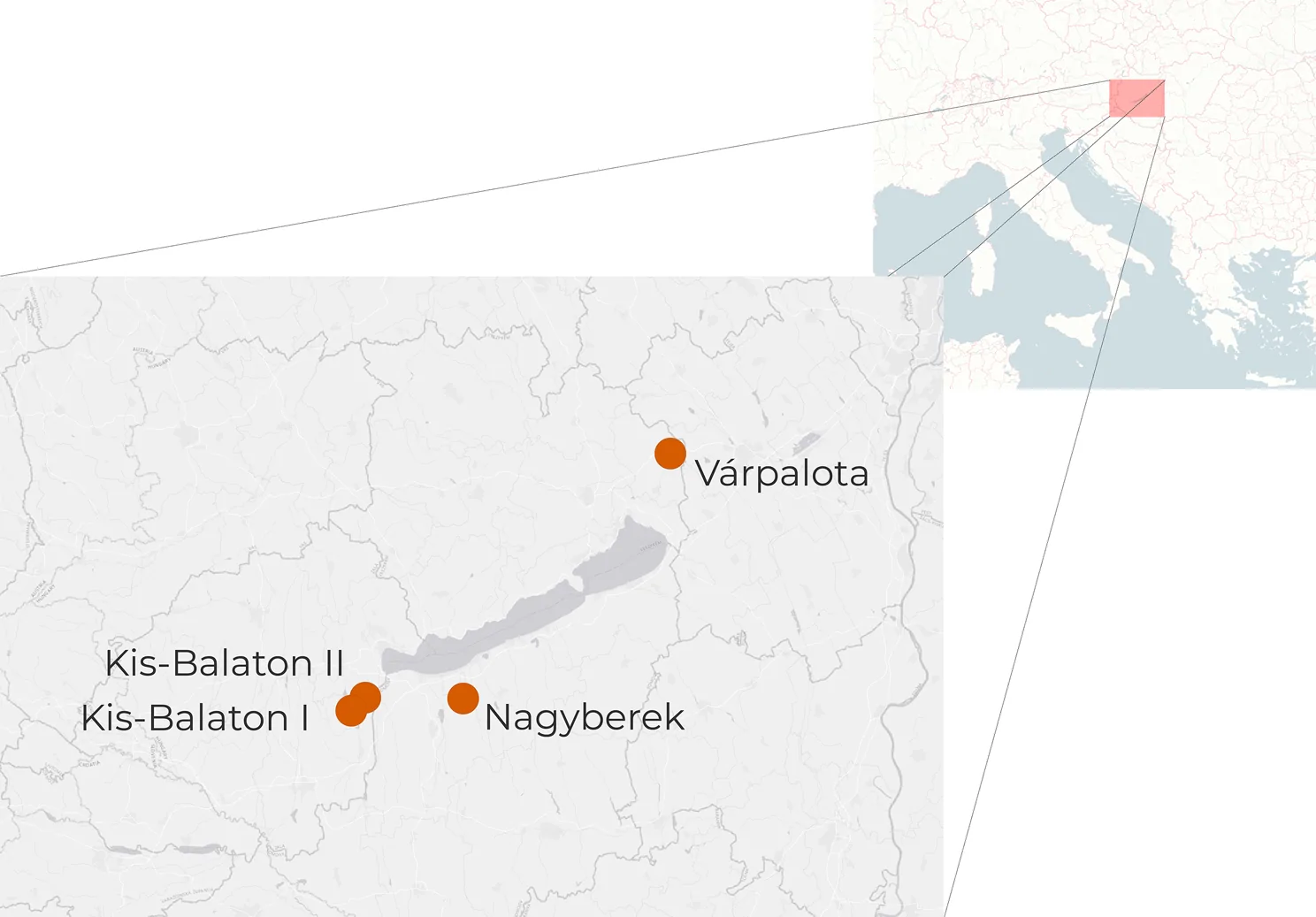
Location of the Great Cormorant breeding colonies around lake Balaton, Hungary. Basemap: Esri, DeLorme, HERE, MapmyIndia

**Fig. 2:**
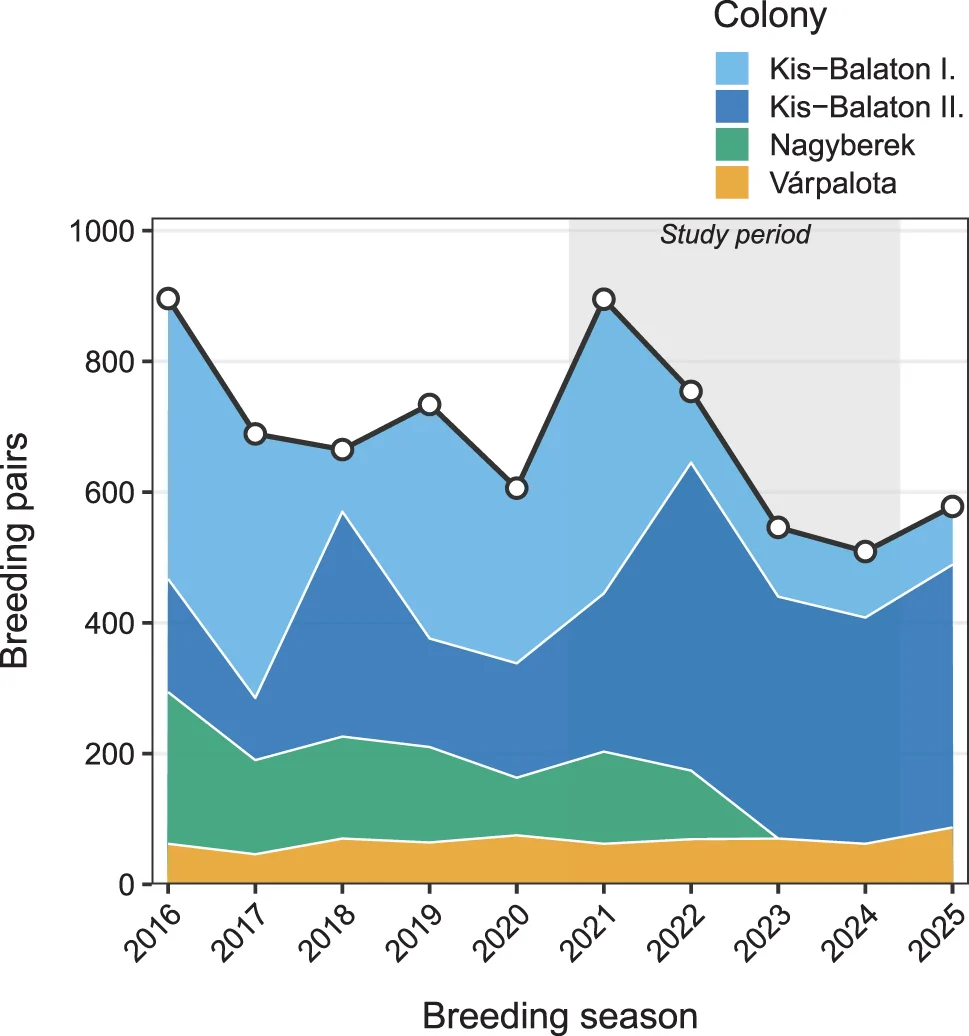
Breeding population of Great Cormorants on all known breeding sites around Lake Balaton, Hungary. This breeding population showed a marginally non-significant negative trend over this period. Extinction of Nagyberek colony is unlikely to be related to our tagging activity, since this location was only used for tagging in the breeding season of 2021, and in the subsequent year the colony still existed

Lake Balaton is the largest Central European lake located in Hungary (Fig. 1). The lake and its adjacent wetlands are protected under the EU Birds and Habitats Directives (Directive 2009/147/EC; Directive 92/43/EEC) and are designated areas under the Ramsar Convention as wetlands of international importance for waterfowl habitat (Ramsar Convention, 1971). Although the lake and the wetlands are included in a mosaic of agricultural and holiday areas, several areas are also under national protection. The Great Cormorant population is spread across four breeding colonies, only one of these colonies is located outside the protected areas. The breeding population in the wetlands surrounding Lake Balaton fluctuated between 509 and 896 pairs during the breeding seasons of 2016–2025 (Fig. 2, mean ± SD: 687 ± 135 pairs), with a non-significant negative trend over the study period (linear regression: slope = −26.4 pairs/year, R^2^ = 0.35, *p* = 0.072; Mann-Kendall test: Z = −1.79, *p* = 0.074), and shows a dynamic variation between years in distribution across the colonies [20]. According to ringing data individuals of this population show southward dispersion and migration movement towards the western Balkan Peninsula and the Central Mediterranean region, while overwintering individuals mix with wintering population arriving mainly from the Baltic region [21]. Tagging of birds was carried out at the three colonies within the protected wetlands under the conservation management of Balaton-felvidéki National Park Directorate (Fig. 1), Kis-Balaton I (46°37’30.5“N 17°08’48.8“E), Kis-Balaton II (46°39’18.1“N 17°11’31.7“E), and Nagyberek (46°39’02.6“N 17°30’23.0“E). All three are tree-nesting colonies; trees of Kis-Balaton I and Nagyberek are on islands, whereas at the colony of Kis-Balaton II nests are in dead trees standing in water since the previously pasture area was flooded for conservation reasons (Figs. S1 and S2).

### Capture and tagging

Sampling design aimed for tagging 10 adult and 10 juvenile individuals. After initial success with capturing adults, it became increasingly difficult, and concerns were raised for causing too much disturbance at the colony. Therefore, after unsuccessful capture attempts of adults pre-fledging nestlings were given priority to minimise disturbance. Devices were deployed on 5 adults (three females and two males, sexing based on biometric data following [22]) and 19 fledglings across 2021–2023 (Table S1).

Adult cormorants were captured on the nest during the chick rearing period within two weeks of hatching using remotely operated spring traps (Video S1). Nestlings were caught by hand before anticipated fledging. All birds were ringed on their left leg with a uniquely numbered metal ring from the Hungarian ringing scheme and additionally on their right leg with a plastic ring with a unique combination of colour and inscription. Transmitters (see specifications below) were attached using a backpack with Teflon ribbon harness with a neck and body loop following Lameris et al. [23], with total package weight (<30 g) remaining well below 3% of body mass (mean * ± * SE in the population 2246 * ± * 74 g, *n* = 15 adults, unpublished own data) to comply with widely accepted bio-logging guidelines. Although transmitter mass in this study remained well below widely accepted bio-logging thresholds, short-term deployments within such limits have been shown to reduce flight time by approximately 30% in breeding seabirds, without detectable effects on breeding success or survival [24]. GPS-derived time budgets should therefore be interpreted with this caveat in mind. Any underrepresentation of flight activity would, however, be conservative with respect to our primary focus on foraging habitat use rather than movement intensity. Few of the transmitters were recovered and redeployed after the decease of tagged individuals.

### Transmitter specifications

Solar-powered GPS transmitters (model: OrniTrack-20; weight: 24 g) recorded locations at dynamically changing intervals. We aimed to record a GPS fix in every 10 minutes, however e.g. upon low battery levels due to inefficient charging during winter months or battery age devices automatically changed fixing routine, and in some cases, we manually reconfigured the settings according to life history stage of the bird, light conditions, charging performance of the devices, and battery status to extend battery life. Fix intervals closely matched the 10-min target across all seasons (median: 10 min). The 95th percentile was marginally elevated in winter (20.7 min) relative to the breeding (15.0 min) and post-breeding (15.5 min) seasons, likely reflecting intermittent data gaps caused by reduced solar charging under low-irradiance conditions. Overall device performance remained stable throughout the study period. Data were transmitted via GSM and stored at GLOSENDAS (glosendas.net), and in Movebank (movebank.org) servers. Each device recorded UTC date & time, GPS position, GPS altitude, speed, direction, horizontal dilution of precision, battery voltage, battery charging current, instant acceleration (3 axes), temperature, magnetic field strength (3 axes). Mean deployment duration per individual was 190 days (SD = 147, median = 164 days, range: 34–628 days, *n* = 24 deployments), reflecting considerable variation, largely attributable to early cessation mainly attributable to mortality events (Table S1).

### GPS accuracy assessment

To verify positional accuracy under local conditions, stationary tests were performed at three reference points across different habitats (urban, gallery forest, lakeshore), day and night conditions, four seasons, and across years 2021–2025. Positional error was quantified as the Euclidean distance between each recorded position and the centroid of all fixes in the respective stationary test. Mean horizontal error and 95% confidence intervals were computed separately for the three habitats. This approach estimates positional precision rather than absolute accuracy, as no independent high-precision reference measurements were obtained at the test locations. Stationary tests (8 tests, 3,694 fixes) yielded a mean positional error of 24.6 ± 8.8 m (SD), with habitat-specific means ranging from 19.0 m (lakeshore) to 42.6 m (gallery forest). Given the 500 × 500 m grid resolution used in habitat classification (see *Habitat classification* section below), even the largest observed errors represent less than 9% of the cell edge length, and are therefore unlikely to result in systematic misclassification of foraging locations (for more details see Supporting Information).

### Data filtering

For adults, data was used from midnight following deployment to account for the effects of handling derived stress on individual behaviour. For fledglings, data was used from midnight preceding the onset of independence (for details see Supporting Information). In all cases data were used until the midnight preceding the cessation of data transmission (in case of device failure) or the day of the individual’s death (based on confirmed mortality event or on movement behaviour). This yielded a total of 418,620 GPS fixes.

In subsequent analyses, locations with dilution of precision (DOP) values exceeding 2, and number of satellites used to determine corresponding GPS position (satcount) below 4 (7.1% of all fixes) were excluded to ensure location precision. Fixes with unrealistic speed data (>100 km/h) were also excluded to account further for measurement error (in earlier work, max speed for great cormorants were measured below 80 km/h, see [25, 26]).

To assess foraging habitat use of tagged birds, we used only daytime data (64.3% of precision filtered data). Daytime was determined as the period from sunrise to sunset at the GPS locations and the corresponding timestamp of the GPS fixes. To ensure spatial representativity and robust estimates for habitat use, we excluded three individuals with fewer than 500 daylight GPS locations from habitat choice analyses. This conservative threshold accounts for temporal autocorrelation in GPS data and ensures stable utilization distribution estimates [27]. For included individuals, number of daylight fixes ranged from 2,225 to 41,843 locations (mean * ± * SD = 11,862 * ± * 9,633, *n* = 21, a total of 249,097 fixes).

### Behaviour classification

Movement behaviour was classified into four categories based on speed (km/h) recorded for each GPS fix: stationary (<2), foraging (2–10), commuting (10–30), and in flight (>30) based on Yoda et al. [26] – for more details see Supporting Information. We used these categories to calculate individual time budgets. In subsequent analysis of foraging habitat use we used individual fixes with the corresponding speed category ranging from 233 to 2,859 fixes (mean * ± * SD = 987 * ± * 745, *n* = 21, a total of 20,678 fixes).

### Habitat classification

We identified 293 high-intensity 500 × 500 m foraging core hotspots from the foraging GPS fixes while ensuring representation of all 21 individuals. Spatial autocorrelation of foraging fix density was quantified using the Getis-Ord Gi statistic (R package *spdep* v1.4–2 [28]); with k = 8 nearest-neighbour spatial weights. No distance-based spatial filter was applied; however, all identified core hotspot cells fell within 210 km of the nearest breeding colony and were classified exclusively as inland freshwater habitats, with no coastal or marine habitat types represented in the hotspot network (Fig. S3). To capture habitat continuity around core hotspot cells (*n* = 300), we identified buffer cells (*n* = 1,125) around each core hotspot cells or clusters of core hotspot cells. This approach ensured that areas in the immediate vicinity of high-use foraging zones were included, recognizing that aquatic habitats often extend beyond single 500 × 500 m grid cells, note however that spatial proximity assignment of buffer zones may not accurately reflect habitat type at boundaries between distinct habitat categories. Core hotspots captured 62.0% of foraging GPS fixes, while core and buffer cells together (henceforth collectively ‘hotspots’) captured 72.0%, indicating that the majority of foraging activity occurred within or immediately adjacent to identified hotspots (for more details of hotspot identification see Supporting Information).

Two experienced observers (BP, PS) independently classified each core hotspot cells into 10 aquatic habitat categories using high-resolution satellite imagery (Esri WorldImagery, 0.3 m resolution) in an interactive web interface. Habitat categories were defined based on field observations of cormorant habitat use in the study area, including fishponds, rivers and lakes (see details in Supporting Information). Observers were blinded to each other’s classifications and for individual bird identity or foraging intensity to minimize bias. Fixes within core hotspot cells were assigned to the corresponding habitat type, while fixes in buffer zones were assigned to the habitat type of the nearest core hotspot via spatial proximity.

### Statistical analysis

To characterise individual variation in foraging habitat use, we constructed an individual × habitat matrix in which each cell represented the proportion of foraging GPS fixes assigned to a given habitat type within an individual’s classified hotspot network. The ten original habitat categories were consolidated to six prior to analysis: ‘reservoir’ and ‘canal’ were merged into ‘regulated water body’, and ‘urban water body’, ‘mixed/uncertain’, and ‘other’ contained no foraging fixes. Analyses were based on seasonal habitat use profiles, calculated separately for each individual × calendar season combination (Spring: March–May; Summer: June–August; Autumn: September–November; Winter: December–February), retaining only combinations with ≥10 foraging fixes (for individual-level seasonal fix counts and year coverage, see Table S2). For immature individuals, data commenced from the onset of independent movement (see *Data filtering* section above).

Among-individual differences in habitat use were quantified using permutation-based multivariate analysis of variance (PERMANOVA [29]); on Bray–Curtis dissimilarities computed from seasonal habitat use profiles (*vegan* v2.7-2 [30]), with individual identity and age class as explanatory terms (marginal effects, 9,999 permutations). Homogeneity of multivariate dispersions among age classes was verified using PERMDISP (*betadisper*, 9,999 permutations) prior to interpreting PERMANOVA results. Between- versus within-individual dissimilarity structure was further examined using analysis of similarities (ANOSIM [31]). Principal coordinates analysis (PCoA) on Bray–Curtis dissimilarities computed from the overall (non-seasonal) individual habitat use matrix was used to visualise individual-level variation in habitat use; associations between habitat type vectors and ordination axes were assessed by permutation tests (*envfit*, 9,999 permutations).

Temporal consistency in individual habitat specialisation was quantified by estimating adjusted repeatability (*R*) for each habitat type separately using linear mixed models with a Gaussian error distribution (*rptR* v0.9.23 [32]). In each model, the proportion of seasonal fixes in a given habitat type was the response variable; individual identity was included as a random effect and age class as a fixed effect. Significance was assessed by likelihood ratio tests (LRT); empirical 95% confidence intervals were obtained by parametric bootstrapping (1,000 iterations) and corroborated by permutation tests (1,000 permutations). The Gaussian approximation was justified because proportions were derived from seasonal fix counts consistently exceeding the minimum threshold (≥10 fixes per season), following Nakagawa & Schielzeth [33].

All analyses were performed in R v4.5.2 [34]. Spatial data were processed with *sf* (v1.0-24 [35]). Data manipulation and visualisation used *tidyverse* (v2.0.0 [36]), *lubridate* (v1.9.4 [37]), and *ggplot2* (v4.0.1 [38]). Ordination was performed with *vegan* (v2.7-2 [30]). Inter-observer agreement was quantified using *irr* (v0.84.1 [39]), repeatability with *rptR* (v0.9.23 [32]), and accelerometer validation with *caret* (v7.0-1 [40], and *corrplot* (v0.95 [41]).

## Results

### Time budget

The 21 GPS-tracked individuals contributed 249,097 daytime fixes in total (mean ± SD per individual: 11,862 ± 9633). Most daytime locations were classified as stationary (85.9 ± 2.7%), with foraging accounting for 8.6 ± 2.0%, in-flight movement for 4.5 ± 1.7%, and commuting for 1.0 ± 0.3%. Time budget proportions varied among individuals: foraging ranged from 4.9% to 12.5%, and flight from 1.1% to 7.8% (Table S3).

### Inter-observer agreement and individual variation in habitat use

Habitat classification agreement between observers was high (exact agreement: 89.1%; *n* = 293 core hotspot cells). Cohen’s κ = 0.833 indicates strong agreement beyond chance [42]. The 32 discrepant cells (10.9%) were resolved by consensus before analysis.

**Fig. 3:**
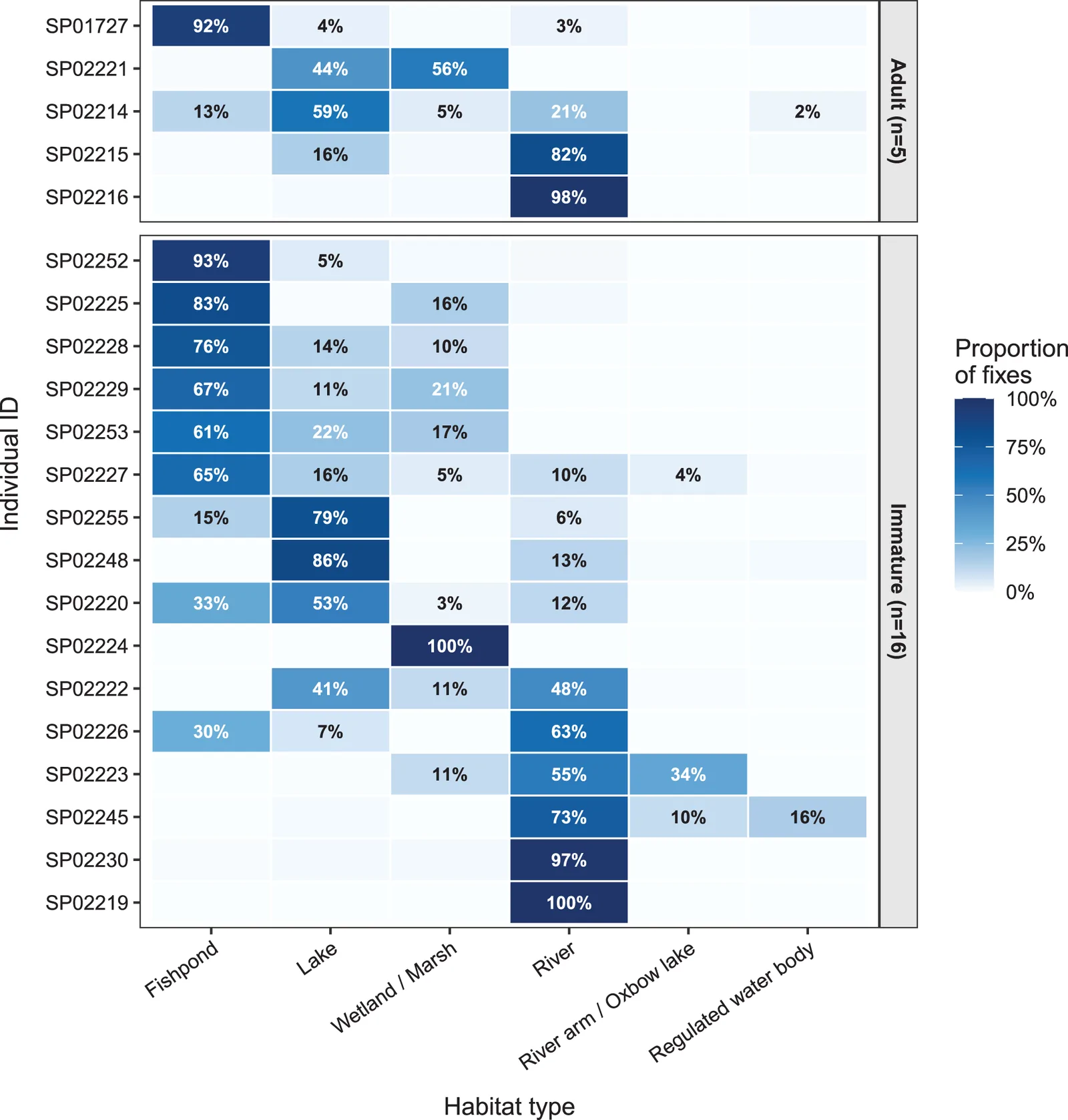
Individual foraging habitat use profiles of GPS-tracked Great Cormorants (*n* = 21) at Lake Balaton and its surroundings, Hungary (2021–2023). Each cell shows the proportion of foraging GPS fixes assigned to a given habitat type within the individual’s classified hotspot network. Only proportions ≥ 2% are labelled. Individuals are grouped by age class and ordered by dominant habitat type within each group. Habitat classification was based on Getis-Ord G*i hotspot analysis combined with manual landscape verification (inter-observer agreement κ = 0.847)

Individual foraging habitat use profiles varied markedly across the population (Fig. 3). Age class explained a negligible and non-significant fraction of this variation (Table 1), and multivariate dispersion did not differ between age classes (PERMDISP: F1,19 = 0.008, *p* = 0.929), confirming that the null result reflects a genuine absence of centroid differences rather than variance homogeneity masking them. By contrast, individual identity accounted for 73.6% of total variation in seasonal foraging habitat use profiles, and between-individual dissimilarities significantly exceeded within-individual dissimilarities, jointly indicating strong, consistent individual-level habitat specialisation (Table 1).

**Table 1:** Results of multivariate tests for individual variation in foraging habitat use

Source of variation	d.f.	SS	R^2^	F	p
***PERMANOVA – age class effect (overall individual × habitat matrix)***					
Age class	1	0.066	0.012	0.235	0.887
Residual	19	5.304	0.988	—	
Total	20	5.370	1.000	—	
***PERMANOVA – individual identity effect (seasonal individual × habitat matrix)***					
Individual identity	19	10.995	0.736	4.902	<0.0001
Residual	32	3.778	0.253	—	
Total	52	14.932	1.000	—	
***ANOSIM – between- vs. within-individual dissimilarity (seasonal matrix)***					
Individual identity	—	—	0.511^a^	—	<0.0001

Results of PERMANOVA (*adonis2*, Bray–Curtis dissimilarity, 9,999 permutations, marginal effects) and ANOSIM testing for (a) the effect of age class on overall individual foraging habitat use profiles (*n* = 21 individuals), and (b) the effect of individual identity on seasonal habitat use profiles (*n* = 53 individual × season observations, *n* = 21 individuals)

SS: sum of squares; R^2^: proportion of total variation explained;—: not applicable

^a^ ANOSIM R statistic (not R^2^); ranges from 0 (no separation) to 1 (complete separation between individuals)

**Fig. 4:**
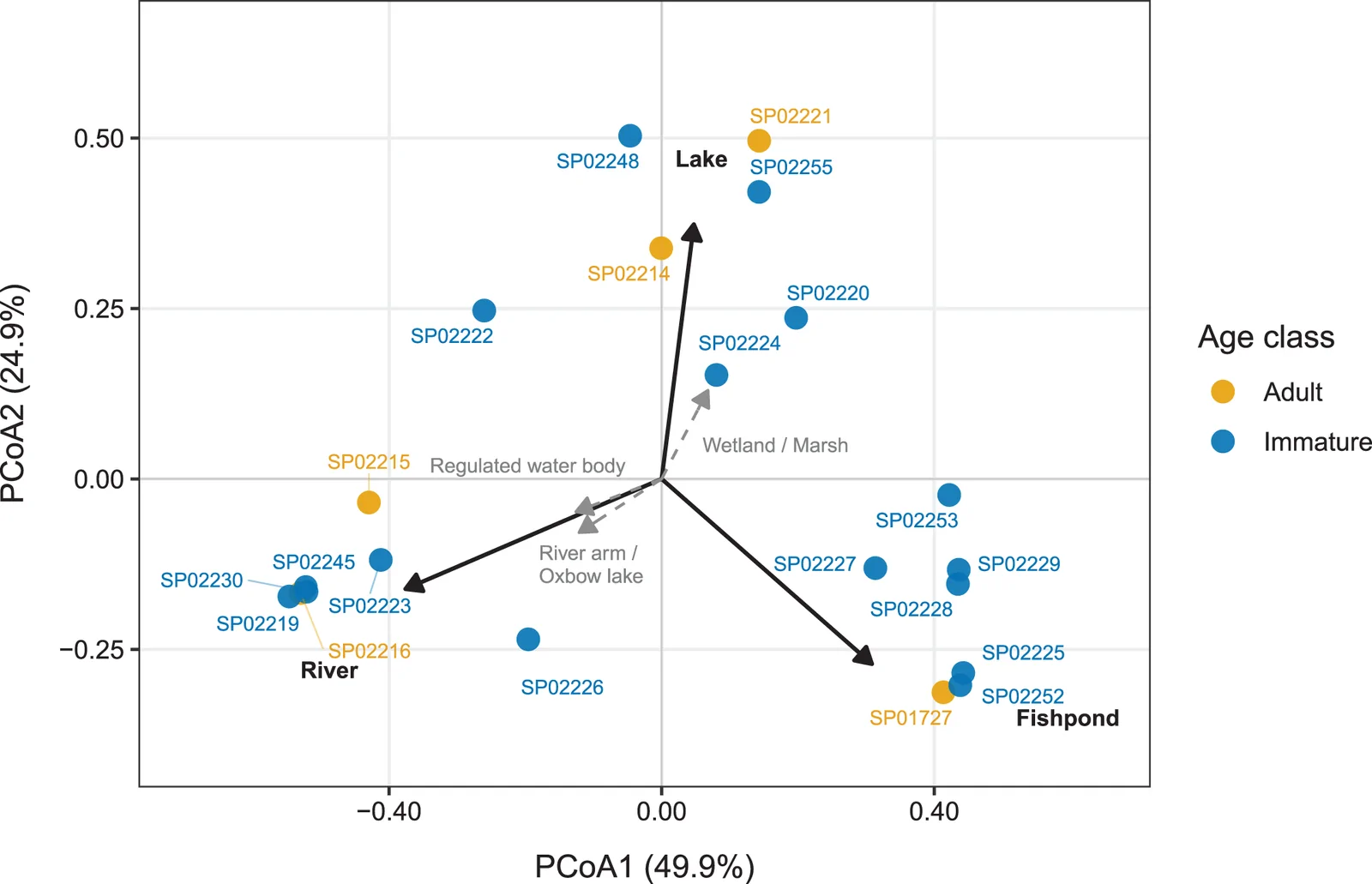
Principal coordinates analysis (PCoA) of individual foraging habitat use profiles of Great Cormorants based on Bray-Curtis dissimilarities (*n* = 21 individuals). Each point represents one individual; point colour indicates age class. Arrows show habitat vectors fitted by permutation test (*envfit*, 9,999 permutations): solid arrows indicate significant associations (*p* < 0.05), dashed arrows indicate non-significant associations (*p* ≥ 0.05). Arrow length is proportional to the strength of association (r^2^). The first two PCoA axes explained 74.8% of total variation in individual habitat use profiles

PCoA ordination confirmed this structure: The first two axes captured 74.8% of total variation (PCoA1: 49.9%, PCoA2: 24.9%; Fig. 4). Fishpond, river, and lake use were the primary axes of differentiation among individuals, with all three habitat vectors strongly associated with the ordination space (fishpond: r^2^ = 0.957, *p* < 0.001; river: r^2^ = 0.949, *p* < 0.001; lake: r^2^ = 0.803, *p* < 0.001). Vectors for wetland/marsh, river arm/oxbow lake, and regulated water body were not significantly associated with either axis (*p* ≥ 0.05), indicating that variation in these habitat types contributed little to individual differentiation in the population.

### Temporal consistency of individual habitat specialisation

**Fig. 5:**
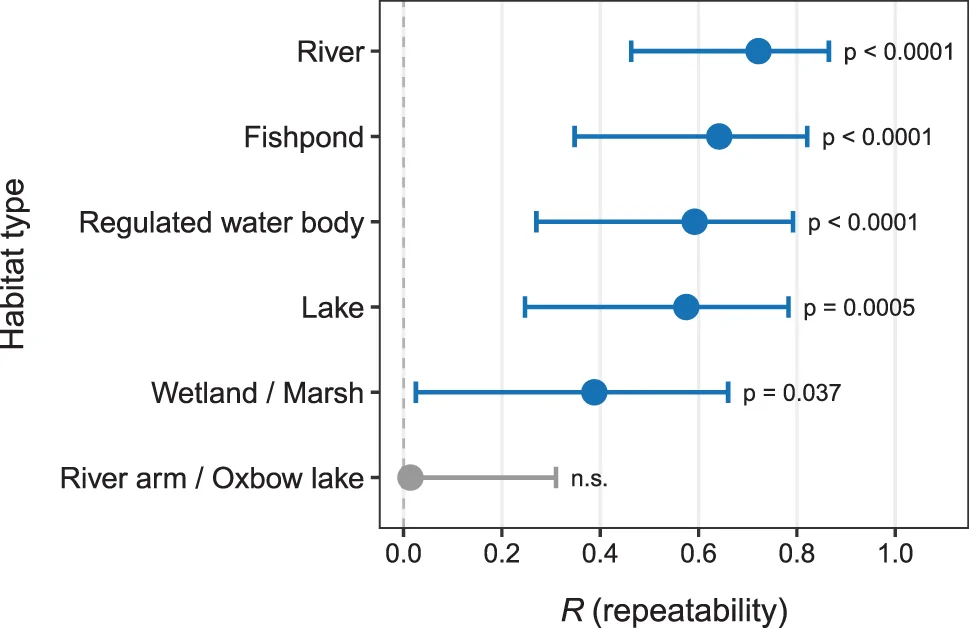
Repeatability of individual foraging habitat use of Great Cormorants across seasons. Points show adjusted repeatability estimates (*R*) with 95% empirical bootstrap confidence intervals (1,000 iterations) derived from linear mixed models (Gaussian distribution) with age class as a fixed effect (*n* = 17 individuals, 49 seasonal observations per habitat type). Significance was assessed by likelihood ratio tests (LRT); blue: significant (*p* < 0.05), grey: non-significant. Habitat types are ordered by decreasing *R*

Foraging habitat use was significantly repeatable across seasons for four of the six habitat types (Fig. 5 and Table 2). Repeatability was highest for rivers and fishponds, with regulated water bodies and lakes showing comparably strong consistency. Wetland/marsh use was repeatable but substantially weaker. River arm/oxbow lake use showed no significant repeatability, a result most plausibly attributable to the rarity of this habitat at the population level – only 2 of 21 individuals used it substantially – precluding reliable estimation of among-individual variance (Table 2).

**Table 2:** Repeatability of individual foraging habitat use across seasons

Habitat type	R	95% CI lower	95% CI upper	*p***(LRT)**
River	0.722	0.463	0.865	<0.001
Fishpond	0.642	0.348	0.821	<0.001
Regulated water body	0.592	0.270	0.792	<0.001
Lake	0.575	0.247	0.783	<0.001
Wetland/Marsh	0.388	0.025	0.660	0.037
River arm/Oxbow lake	0.014	0.000	0.310	1.000

Adjusted repeatability (*R*) of the proportion of seasonal foraging fixes assigned to each habitat type, estimated using linear mixed models (Gaussian distribution) with individual identity as a random effect and age class as a fixed effect (*n* = 17 individuals, 49 seasonal observations per habitat type). Confidence intervals were obtained by parametric bootstrapping (1,000 iterations); significance was assessed by likelihood ratio tests (LRT). *R* ranges from 0 (no among-individual variation) to 1 (all variation among individuals). Habitat types are ordered by decreasing *R*

## Discussion

Within the studied inland-breeding Great Cormorant population, individual identity emerges as the dominant structuring force of foraging habitat use—specialisation that persists across seasons and operates independently of age class.

The magnitude of among-individual variation documented here – individual identity explaining nearly three-quarters of total variation – substantially exceeds benchmarks from the broader behavioural ecology literature. Bell et al. [43] report a mean repeatability of *r* = 0.37 across 759 estimates spanning diverse behaviours and taxa, with habitat selection among the most repeatable behavioural classes. Our adjusted *R* values of 0.575–0.722 not only surpass this mean but approach the upper range reported for habitat selection in vertebrates. Within the Phalacrocoracidae, GPS tracking has consistently revealed strong foraging-site fidelity across species and contexts [12, 16, 17], yet these studies typically followed few individuals over a single season, precluding any quantification of temporal consistency. Harris et al. [13] provided the most directly comparable evidence, documenting multi-season consistency in Imperial Shag foraging behaviour; our study extends this finding to an inland-breeding *Ph. c. sinensis* population, across a broader set of ecologically distinct habitat types and a larger sample of individuals. Potier et al. [44] quantified individual repeatability of foraging behaviour in Great Cormorants at a coastal colony. At the population level, departure angle and trip duration were among the most repeatable variables, yet these indices concealed wide variation in the degree of consistency among individuals – some birds were persistently flexible, others highly consistent. Trip-level consistency was positively associated with foraging efficiency, suggesting that the high habitat-use repeatability we document here may yield fitness benefits through accumulated knowledge of, and reliable access to, predictable prey patches. The parallel between trip-level consistency and foraging efficiency in Potier et al. [44] and the high habitat-use repeatability documented here suggests that stable habitat specialisation may be maintained, at least in part, through fitness advantages from site familiarity and predictable prey access. Individual consistency in habitat use across seasons is not unique to cormorants. Hoye et al. [45] showed persistent individual specialisation along a terrestrial–aquatic gradient in migrating Bewick’s swans, with carry-over effects on body condition and subsequent reproductive success—underscoring that habitat-use repeatability can carry fitness consequences beyond the immediate foraging context.

The ecological framework of Araújo et al. [10] identifies intraspecific competition, habitat predictability and individual learning as the principal drivers of individual specialisation. In the human-modified freshwater landscape surrounding Lake Balaton – a mosaic of intensively managed fishponds, regulated rivers and large natural lakes – these drivers plausibly act synergistically. Fishponds are spatially discrete, temporally predictable prey patches with high fish density; once an individual establishes profitable foraging routines at such a site, both accumulated site familiarity and competitive exclusion by conspecifics may reinforce specialisation across seasons [10]. Rivers and lakes are structurally less predictable and likely favour divergent foraging strategies. That both fishpond use and river use show strong repeatability suggests these reinforcing mechanisms operate independently on distinct habitat types within the same population.

PCoA ordination revealed a clear three-way structure in individual habitat use with fishpond, river, and lake use constituting the three dominant and largely independent axes of differentiation. Age class explained a negligible fraction of this variation, and multivariate dispersion did not differ between age classes – indicating that habitat specialisation is not driven primarily by ontogenetic niche shifts. This contrasts with the common expectation that juveniles should exploit habitats more opportunistically before settling into a specialised niche as adults [10]. One plausible explanation is that specialisation is established early, during the first post-fledging season, when young birds explore and select among available freshwater habitats – a period captured in detail here by tracking individuals from the onset of independence.

The absence of detectable age-class effects also bears on the interpretation of the underlying mechanisms. In coastal *Ph. c. sinensis*, Fijn et al. [7] documented sex-specific habitat segregation, with males largely avoiding freshwater; the authors attributed this to competitive displacement of females from nearshore marine foraging sites. In our inland population, this dynamic operates on a different axis: Because all individuals are constrained to freshwater, differentiation occurs among freshwater habitat types rather than between habitat domains. The consistent use of distinct freshwater types by different colony members points to intraspecific niche partitioning that reduces exploitative competition without spatial exclusion from an entirely different environment. Bearhop et al. [15] demonstrated analogous long-term individual specialisation and sex-specific niche partitioning in closely related shags via stable isotopes; that equivalent patterns are detectable with GPS tracking across discrete freshwater habitat types underscores the complementarity of the two approaches.

Previous GPS tracking studies of Great Cormorants have predominantly focused on coastal or estuarine colonies [7, 14, 26, 44], and the limited tracking data available for inland-foraging birds derive from coastal colonies, covering too few individuals to support robust quantification of among-individual variation. To our knowledge, this is the first study to formally quantify both the magnitude and temporal consistency of foraging habitat specialisation across ecologically distinct freshwater habitat types in an inland-breeding population of *Ph. carbo*. The repeatability values reported here may reflect the particularly structured nature of anthropogenic freshwater landscapes, where discrete, managed habitat patches such as fishponds offer unusually stable and predictable foraging opportunities relative to open marine systems. Cole et al. [9] showed that Great Cormorants in tidal waters select for fine-scale hydrodynamic conditions with high precision; in our system, the equivalent selective gradient operates at the landscape scale, expressed as differences in management regime, water body size and fish community composition among fishponds, rivers and lakes. Foraging-site fidelity in Great Cormorants has been shown to be robust even to varying wind conditions [26], reinforcing the notion that individual habitat choice reflects stable, intrinsically motivated preferences rather than opportunistic responses to short-term environmental variation.

The individual habitat fidelity documented here persists into winter: Marked birds from other populations also showed high site fidelity, with mean stays of 64 days [46] – underscoring the seasonal stability of habitat use. This parallels the ‘problem individual’ paradigm [3, 19], in which targeting high-impact specialists yields greater management returns than indiscriminate population-level action. Ovegård et al. [47] found that cormorant predation effects on fish populations vary markedly among prey taxa and management contexts – heterogeneity that our results suggest could partly be driven by among-individual variation in habitat use within populations.

### Limitations and future directions

With 21 tracked individuals, repeatability estimates carry substantial uncertainty, and the sample is too small for formal modelling of individual-level predictors of specialisation such as sex, body condition or colony of origin. The imbalance between tagged fledglings (*n* = 19) and adults (*n* = 5) is a particular constraint: Sex-specific differences in habitat specialisation – well-documented in coastal populations [7] – could not be tested here. A sex-balanced sample in future work would allow direct testing of whether the intraspecific competition mechanisms described by Fijn et al. [7] also structure differentiation among freshwater habitat types. The behaviour classification used here – based on GPS-derived speed thresholds – reliably identifies foraging locations but cannot resolve dive effort or prey capture rate (see Supporting Information). Combining high-frequency accelerometry with GPS tracking would enable quantification of individual differences not only in where birds forage, but in the intensity and efficiency of foraging within each habitat type – a distinction with direct relevance to estimating individual-level predation impact on fish stocks.

## Conclusions

GPS tracking in an inland-breeding Great Cormorant population at Lake Balaton showed that individual identity, not age class or opportunistic habitat switching, drives foraging habitat use. Individual identity accounted for 73.6% of total variation in seasonal habitat use profiles; repeatability was high for four of six freshwater habitat types (*R* = 0.575–0.722), with fishponds and rivers showing the strongest consistency. Principal coordinates analysis identified fishpond, river and lake use as three largely independent axes of individual differentiation – the population comprises a structured assemblage of persistent habitat specialists, not a behaviourally homogeneous unit.

Habitat partitioning in this inland population is not an artefact of ontogenetic niche shifts or short-term environmental responses. It emerges from stable, individually acquired movement tactics maintained across seasons. The degree of specialisation reported here – approaching the upper range for habitat selection across vertebrates – places inland-breeding Great Cormorants among the most consistent individual habitat specialists documented in colonial waterbirds. That this consistency is already present in juveniles tracked from the onset of independence suggests habitat preferences are established early, then reinforced likely through site familiarity and predictable prey availability.

The implications for wildlife–fishery conflict management are direct. Assuming uniform habitat exploitation across a colony, population-level interventions will produce disproportionate, habitat-sector-specific outcomes. Individuals that consistently forage in fishponds generate concentrated, predictable predation pressure on managed stocks; conspecifics using rivers or lakes exert qualitatively different impacts – and the relative severity of these impacts reverses depending on whether management priorities concern commercial aquaculture or recreational and artisanal fisheries in natural water bodies. Applying the ‘problem individual’ logic to an avian piscivore, these results support moving from indiscriminate population-level control toward habitat-explicit strategies. Incorporating individual tracking data results into conflict assessments and risk-mapping frameworks offers a tractable, evidence-based route to reducing cormorant–fishery conflicts.

## Electronic supplementary material

Below is the link to the electronic supplementary material.

Supplementary Material 1

Supplementary Material 2

## Data Availability

Tracking datasets and derived products generated and analysed during the current study are deposited in Movebank [movebank.org, study name “Great Cormorant (*Phalacrocorax carbo*) research in the Carpathian Basin”, study ID 2275792407, and study name “Great cormorant (*Phalacrocorax carbo*) research at Lake Balaton”, study ID 1484473813], and will be archived in the ARP Research Data Repository [48] under handle ID 21.15109/ARP/VSWOGR upon publication. GPS data will be openly accessible three years after publication to provide the data creators the opportunity of first use of the data in other analyses; all other files will be available upon publication. We are committed to open data sharing and will make the full dataset openly accessible upon expiry of the embargo. In the interim, access may be granted by the corresponding author upon reasonable request.
