# Supplementary material for: Consistent individual differences in foraging movement tactics drive habitat partitioning in an inland-breeding Great Cormorant population
Source: Mov Ecol. 2026 Jul 21;14:71. doi: 10.1186/s40462-026-00682-6 (PMC13673635; doi:10.1186/s40462-026-00682-6)
Supplement: Supplementary file 1 [file 40462_2026_682_MOESM2_ESM.pdf]

## Supporting Information for

# Consistent individual differences in foraging movement tactics drive habitat partitioning in an inland-breeding Great Cormorant population

Preiszner, B.*, Szinai, P. & Kovács, Gy.

* preiszner.balint@blki.hu

## GPS Accuracy Assessment

Eight stationary accuracy tests (3,694 GPS fixes in total) were conducted across three habitat types. Overall positional error was 24.6 ± 8.8 m (mean ± SD), with individual test means ranging from 17.7 to 42.6 m. Lakeshore habitat yielded the highest positional accuracy (19.0 ± 1.5 m; 5 tests, 824 fixes), followed by urban environment (29.5 ± 1.7 m; 2 tests, 2,582 fixes), while a single gallery forest test produced the largest error (42.6 m; 288 fixes). The reduced precision in forested and urban settings is consistent with signal attenuation and multipath effects attributable to canopy cover and structural obstructions, respectively — though this interpretation is based on contextual inference rather than direct measurement of sky visibility or signal quality. Open-water foraging habitat, the primary focus of this study, structurally resembles the lakeshore test conditions; positional error in this habitat type is therefore expected to fall within the lower range of observed values (approximately 17–20 m), though this remains an assumption that could not be independently verified under foraging conditions.

## Data filtering

In fledglings, first departure from nest (henceforth fledging event) was determined based on GPS data. First, the baseline centroid was calculated from the first 3 days of GPS data following logger deployment, filtered for quality (HDOP<3) and outliers (>95th percentile distance removed). Three-day period was chosen based on colony observations as it represents the anticipated minimal nest-bound period for fledglings at the size suitable for tagging. A fledging event was defined as two consecutive GPS fixes both exceeding 100 m from the centroid. This threshold is more than 3-fold the mean positional error observed in stationary tests (see Methods of main text), ensuring a conservative detection criterion that minimizes false positives from GPS fix error while

detecting meaningful fledging events (see Table S4 to compare with 200 m threshold). Although 10 individuals' data showed fledging events during the three-day baseline period, this did not change the data qualitatively. We used 4 different settings to assess sensitivity, and this conservative combination yielded the most comprehensive data (Table S4). After defining fledging events, we defined the onset of independent movement as the first of two consecutive days with a minimum of 5 fledging events each. Independence started 27.16 $\pm$ 2.89 (mean $\pm$ SE) days after first fledging event (Table S5; and see individual figures in Preiszner et al., 2026). We consider this as a conservative approach to assess foraging habitat use, since in the closely related double-crested cormorant (*Nannopterum auritum*), post-fledging parental care averages 15.9 days, with fledglings regularly fed at nests during this dependency period (Fraser, 2024).

## Behaviour classification

We validated behavioural classification using tri-axial accelerometer data from GPS fixes. Acceleration differed among classes (Kruskal–Wallis $\chi^2$ = 1195.85, df = 3, $p <$ 0.001), but correlation with GPS speed was low (Spearman r = 0.087). This reflects diving gaps in GPS records (Watanabe et al. 2011). Agreement was high for stationary (75%) and flight (100%), lower for foraging. GPS speed underestimates foraging as dives occur underwater (Watanabe et al. 2011). High-frequency accelerometers with depth sensors would detect dives but were unavailable. Since habitat selection was our focus, we kept speed-based classes. 'Foraging' thus includes surface swimming and diving, with dives underrepresented. Surface speed (2–10 km/h) indicates foraging areas. Our accelerometer validation highlighted a known challenge in aquatic bird telemetry: GPS-derived speed underestimates foraging effort because diving events (high body acceleration) occur underwater where GPS signals are unavailable. High-frequency accelerometry (>10 Hz) combined with depth sensors would be required to fully resolve foraging behaviour (Watanabe et al., 2011), but such systems were not available for this study. However, surface swimming speed (2–10 km/h) remains a reliable proxy for areas where foraging occurs, even if it does not capture dive effort directly.

## Foraging hotspot identification

We projected foraging GPS fixes (*n* = 20,678 from 21 birds) to ETRS89-LAEA Europe (EPSG:3035). Fixes were intersected with a 500 × 500 m grid covering all fixes plus 2.5 km buffer. We selected grid cells with ≥5 fixes (*n* = 651 cells, 97.3% of foraging fixes) in two steps to include all individuals and high-use areas. First, we took the top-3 cells per

bird (highest fix density), giving 19 unique cells that covered all 21 birds. Second, we ranked the rest by a score (70% fix density, 30% number of individuals) and picked the top 281 cells. This gave 300 core hotspots covering all birds (mean 75.2% individual coverage, range 35.3–96.3%). Some cells (26.3%, *n* = 79) had fixes from only one bird, showing variation in habitat use. Sensitivity tests with different thresholds (3–10 fixes per cell, 200–500 total cells) confirmed that 300 cells worked best. The 70:30 score weights matched traditional clustering while keeping habitat variety (Table S6). Five core hotspot cells (1.7%) overlapped breeding colonies and two (0.7%) roosting sites on islands or in-water trees (Figs. S1 and S2). Birds occasionally forage close to these sites (own observation). We excluded these cells to focus on off-colony foraging only. At the same time, this approach kept data from short trips in the immediate vicinity of the colony to the neighbouring core cells. To assess whether the exclusion of Stationary fixes introduced a systematic spatial bias, we compared the spatial distribution of Foraging and Stationary fixes at both the population and individual levels. At the population level, the grid-cell-based densities of the two behavioural categories showed moderate spatial correlation (Spearman ρ = 0.56) and overlap (Jaccard index = 0.48). At the individual level, spatial overlap was similarly moderate (mean Jaccard = 0.45, range: 0.28–0.63; mean Spearman ρ = 0.49, range: 0.16–0.77). When Stationary fixes were included alongside Foraging fixes, 71.0% of the originally identified core hotspot cells (208 of 293) were retained among the top 300 most-used grid cells, indicating that the hotspot configuration was largely robust to the exclusion of Stationary fixes. The moderate rather than high overlap between categories is consistent with Stationary fixes representing a broader and more heterogeneous set of behaviours beyond active foraging and supports the use of Foraging fixes alone as a more precise proxy for foraging habitat use.

## Interactive habitat classification protocol

Core foraging hotspots (*n* = 293 after colony and roost exclusion) were classified using a custom Shiny web application interfaced with high-resolution satellite basemaps (Esri WorldImagery, OpenStreetMap). The interface displayed each 500×500m core grid cell centered within a 2-km contextual view, with optional overlays of GPS foraging points (*n* = 20,678) and adjacent buffer zones. Ten habitat categories were defined a priori: fishpond, river, river arm/oxbow lake, lake, wetland/marsh, reservoir, canal, urban water body, mixed/uncertain, other. Two observers, blind to bird identities and each other's classifications, categorized habitats based on the aerial imagery and local knowledge of fisheries management intensity. Observer experience may have contributed subtle bias beyond visual features alone.  Classifications were recorded with optional field notes and timestamps. Upon completion, observers downloaded CSV files that merged for reproducibility analysis. Latter confirmed excellent inter-observer agreement (see main

text), with highest concordance for river (95.5%), wetland/marsh (94.4%), reservoir (92.3%) lake (88.5%), and fishpond (81.5%) habitats. Before statistical analysis, the ten original habitat categories were reduced to six. Reservoir and canal were merged into a single regulated water body category based on their shared hydrological management characteristics. There were no cells classified as urban water body, mixed/uncertain, or other.

## Management implications

The management relevance of individual-level habitat specialisation is further underscored by the population-level context of this study. In contrast to the broadly stable or increasing trend of *P. c. sinensis* populations in Northern Europe (Bregnballe, T. pers. comm.), the breeding population at Lake Balaton showed a marginally non-significant negative trend over the study period. This local decline coincides with a sustained and intensifying programme of lethal control under derogation from the EU Birds Directive, with culling in Hungary averaging 12.037 birds/year (range: 8,867–17,270) over 2013–2023, of which the Lake Balaton catchment accounted for a mean of 1,204 birds/year (range: 652–2,265; approximately 9.7% of the national total, rising from 7.5% in 2014 to 19.2% in 2023). If the declining local population trend is indeed driven in part by this sustained culling pressure, the behavioural structure documented here – where a subset of individuals are persistent fishpond specialists – becomes directly relevant to evaluating the efficacy of lethal interventions: a population-level cull distributed across all habitat types may remove lake and river foragers disproportionately to the economic goal of fisheries mitigation.

## References

Fraser, G. S. (2024). Post-Fledging Parental Care in Tree-Nesting Double-Crested Cormorants (*Nannopterum auritum*). *Waterbirds*, *46*(2-4), 139-145.

Preiszner, Bálint; Kovács, Gyula; Szinai, Péter, 2026, "GPS tracking of great cormorants (Phalacrocorax carbo sinensis) in the Carpathian Basin", https://hdl.handle.net/21.15109/ARP/VSWOGR, ARP, DRAFT VERSION

Watanabe, Y. Y., Takahashi, A., Sato, K., Viviant, M., & Bost, C. A. (2011). Poor flight performance in deep-diving cormorants. *Journal of Experimental Biology*, *214*(3), 412-421.

**Table S1.:** Deployment details of GPS-tracked Great Cormorants (*Ph. c. sinensis n* = 24) at Lake Balaton, Hungary, 2021-2024.

| Individual ID | Transmitter S/N | Tagging date | Last alive GPS data | Days | Tagging location | Age | Sex | Shot | Fate |
|---|---|---|---|---|---|---|---|---|---|
| SP01727 | 216275 | 25.04.23 | 19.04.24 | 360 | Kis-Balaton II | Adult | Female | Unknown | Unknown |
| SP02214 | 216268 | 22.04.22 | 10.01.24 | 628 | Kis-Balaton II | Adult | Male | Unknown | Unknown |
| SP02215 | 216269 | 25.04.22 | 01.01.23 | 251 | Kis-Balaton I | Adult | Male | Unknown | Unknown |
| SP02216 | 216270 | 10.05.22 | 10.11.22 | 184 | Kis-Balaton II | Adult | Female | Unknown | Stopped transmitting during migration at Mediterranean shores of North Africa. |
| SP02221 | 211682 | 17.04.21 | 23.07.21 | 97 | Kis-Balaton I | Adult | Female | Unknown | Unknown |
| SP02219 | 216276 | 23.05.22 | 06.09.22 | 106 | Kis-Balaton I | Fledgling | NA | Yes | Suspected shooting. Culling operator remembered injured bird. |
| SP02220 | 216275 | 23.05.22 | 01.10.22 | 131 | Kis-Balaton I | Fledgling | NA | No | Confirmed death, unknown cause. Remains found. |
| SP02222 | 211683 | 08.05.21 | 06.11.21 | 182 | Nagyberek | Fledgling | NA | Unknown | Suspected death according to fix patterns. |
| SP02223 | 211684 | 08.05.21 | 13.10.21 | 158 | Nagyberek | Fledgling | NA | Yes | Suspected shooting. Remains found. |
| SP02224 | 211685 | 08.05.21 | 04.07.21 | 57 | Nagyberek | Fledgling | NA | Yes | Confirmed shooting. Transmitter retrieved. |
| SP02225 | 211686 | 08.05.21 | 07.01.22 | 244 | Nagyberek | Fledgling | NA | No | Confirmed death. Likely predation. Remains found, transmitter retrieved. |
| SP02226 | 211687 | 08.05.21 | 24.06.22 | 412 | Kis-Balaton I | Fledgling | NA | Yes | Suspected shooting according to fix patterns. |
| SP02227 | 211688 | 08.05.21 | 02.01.22 | 239 | Kis-Balaton I | Fledgling | NA | Unknown | Suspected death according to fix patterns. |
| SP02228 | 211689 | 08.05.21 | 19.08.21 | 103 | Kis-Balaton I | Fledgling | NA | Yes | Suspected shooting according to fix patterns. |

| Individual ID | Transmitter S/N | Tagging date | Last alive GPS data | Days | Tagging location | Age | Sex | Shot | Fate |
|---|---|---|---|---|---|---|---|---|---|
| SP02229 | 211690 | 08.05.21 | 11.07.21 | 64 | Kis-Balaton I | Fledgling | NA | Yes | Suspected shooting according to fix patterns. |
| SP02230 | 211691 | 08.05.21 | 25.10.21 | 170 | Kis-Balaton I | Fledgling | NA | Yes | Suspected shooting according to fix patterns. Transmitter retrieved. |
| SP02245 | 211686 | 23.05.22 | 08.08.23 | 442 | Kis-Balaton I | Fledgling | NA | Unknown | Unknown |
| SP02248 | 216271 | 02.06.22 | 28.11.22 | 179 | Kis-Balaton I | Fledgling | NA | No | Confirmed death. Remains found, no sign of shooting. |
| SP02252 | 211685 | 02.06.22 | 18.10.22 | 138 | Kis-Balaton I | Fledgling | NA | Yes | Confirmed shooting. Transmitter retrieved. |
| SP02253 | 211691 | 16.06.22 | 31.07.22 | 45 | Kis-Balaton I | Fledgling | NA | No | Confirmed death. Likely predation. Remains found. |
| SP02255 | 211685 | 23.05.23 | 28.01.24 | 250 | Kis-Balaton I | Fledgling | NA | Unknown | Unknown |
| SP02217 [a] | 216278 | 18.05.22 | 21.06.22 | 34 | Kis-Balaton II | Fledgling | NA | Yes | Confirmed shooting. Transmitter retrieved. |
| SP02242 [a] | 216274 | 23.05.22 | 15.07.22 | 53 | Kis-Balaton I | Fledgling | NA | No | Suspected death due to collision. |
| SP02250 [a] | 211692 | 02.06.22 | 07.07.22 | 35 | Kis-Balaton I | Fledgling | NA | Yes | Suspected shooting according to fix patterns, shells found at last fix. |

Dates are in DD.MM.YY format. 'Days' indicates the deployment duration from tagging until last alive GPS position. ‘Age’ refers to age class at tagging. NA = not applicable for sex determination in fledglings. 'Shot' indicates shooting mortality.

[a] Three individuals were excluded from analyses due to lack of sufficient data (minimum 500 daylight locations required).

**Table S2.:** Number of foraging GPS fixes and years of data coverage per individual and season for GPS-tracked Great Cormorants (*Ph. C. sinensis*) at Lake Balaton, Hungary (2021–2024).

| Individual ID | Age | Autumn | Spring | Summer | Winter |
|---|---|---|---|---|---|
| SP01727 | Adult | 1 / 419 | 2 / 350 | 1 / 425 | 2 / 572 |
| SP02214 | Adult | 2 / 836 | 2 / 563 | 2 / 977 | 3 / 480 |
| SP02215 | Adult | 1 / 270 | 1 / 170 | 1 / 379 | 1 / 30 |
| SP02216 | Adult | 1 / 500 | 1 / 111 | 1 / 402 | - |
| SP02219 | Fledgling | 1 / 26 | - | 1 / 275 | - |
| SP02220 | Fledgling | 1 / 117 | - | 1 / 158 | - |
| SP02221 | Adult | - | 1 / 160 | 1 / 308 | - |
| SP02222 | Fledgling | 1 / 322 | 1 / 59 | 1 / 757 | - |
| SP02223 | Fledgling | 1 / 206 | - | 1 / 671 | - |
| SP02224 | Fledgling | - | 1 / 17 | 1 / 216 | - |
| SP02225 | Fledgling | 1 / 386 | 1 / 9 | 1 / 354 | 2 / 106 |
| SP02226 | Fledgling | 1 / 417 | 1 / 620 | 2 / 886 | 2 / 380 |
| SP02227 | Fledgling | 1 / 418 | - | 1 / 436 | 2 / 40 |
| SP02228 | Fledgling | - | - | 1 / 399 | - |
| SP02229 | Fledgling | - | - | 1 / 247 | - |
| SP02230 | Fledgling | 1 / 446 | - | 1 / 802 | - |
| SP02245 | Fledgling | 1 / 410 | 1 / 609 | 2 / 1072 | 2 / 201 |
| SP02248 | Fledgling | 1 / 384 | - | 1 / 231 | - |
| SP02252 | Fledgling | 1 / 280 | - | 1 / 540 | - |
| SP02253 | Fledgling | - | - | 1 / 236 | - |
| SP02255 | Fledgling | 1 / 476 | - | 1 / 428 | 2 / 89 |

Values indicate the number of calendar years with data / total number of foraging fixes (speed 2–10 km/h) for each individual–season combination. Individuals excluded from habitat use analyses (< 500 daytime fixes) are marked with superscript a. Seasons: Spring = March–May, Summer = June–August, Autumn = September–November, Winter = December–February. Dashes indicate season–individual combinations with no data.

**Table S3.:** Daytime activity budgets for 24 GPS-tracked Great Cormorants (*Ph. c. sinensis*) at Lake Balaton, Hungary 2021-2024.

| Individual ID | *n* (daylight) | Stationary (%) | Foraging (%) | Commuting (%) | Flight (%) |
|---|---|---|---|---|---|
| SP02214 | 41848 | 86.7 | 6.8 | 1.2 | 5.3 |
| SP02245 | 25074 | 85.9 | 9.1 | 1.0 | 4.1 |
| SP01727 | 24786 | 85.9 | 7.1 | 0.9 | 6.1 |
| SP02226 | 22013 | 82.0 | 10.5 | 1.5 | 6.0 |
| SP02215 | 16092 | 91.3 | 5.3 | 0.6 | 2.8 |
| SP02216 | 14220 | 87.1 | 7.1 | 0.9 | 4.8 |
| SP02227 | 11519 | 83.7 | 7.8 | 1.4 | 7.1 |
| SP02222 | 11152 | 82.3 | 10.2 | 1.2 | 6.3 |
| SP02230 | 9989 | 81.5 | 12.5 | 1.2 | 4.8 |
| SP02225 | 9281 | 81.5 | 9.2 | 1.5 | 7.8 |
| SP02255 | 8947 | 84.8 | 11.1 | 0.8 | 3.3 |
| SP02248 | 8833 | 88.0 | 7.0 | 1.1 | 4.0 |
| SP02223 | 8321 | 86.5 | 10.5 | 0.6 | 2.4 |
| SP02252 | 8022 | 82.9 | 10.2 | 1.0 | 5.8 |
| SP02221 | 6042 | 88.9 | 7.7 | 0.9 | 2.4 |
| SP02228 | 5702 | 88.1 | 7.0 | 1.1 | 3.8 |
| SP02220 | 5590 | 88.3 | 4.9 | 0.6 | 6.2 |
| SP02219 | 3920 | 86.7 | 7.7 | 1.3 | 4.3 |
| SP02229 | 2885 | 86.6 | 8.6 | 1.2 | 3.7 |
| SP02224 | 2636 | 89.3 | 8.8 | 0.7 | 1.1 |
| SP02253 | 2225 | 85.1 | 10.6 | 0.9 | 3.4 |
| SP02242[a] | 486 | 93.4 | 3.5 | 1.0 | 2.1 |
| SP02250[a] | 445 | 91.0 | 4.5 | 1.1 | 3.4 |
| SP02217[a] | 59 | 93.2 | 1.7 | 5.1 | 0.0 |
| Mean ± SD | 11,862 ± 9,633 | 85.9 ± 2.7 | 8.6 ± 2.0 | 1.0 ± 0.3 | 4.5 ± 1.7 |
| Range (min-max) | 2,225-41,848 | 81.5-91.3 | 4.9-12.5 | 0.6-1.5 | 1.1-7.8 |

Summary statistics (mean ± SD, range) are reported for 24 individuals; individuals marked with [a] were excluded from habitat use analyses due to insufficient spatial data (<500 daytime fixes). Proportions do not necessarily sum to 100% due to rounding.

**Table S4.**: Sensitivity for settings in determining first departure from nest after deployment.

| Parameters | Days to departure (Mean ± SD) | Fledging distance in m (Mean ± SD) |
|---|---|---|
| Threshold 100m Baseline 3 days | 3.01 ± 3.14 | 313 ± 197 |
| Threshold 100m Baseline 1 day | 3.00 ± 3.14 | 313 ± 197 |
| Threshold 200m Baseline 3 days | 8.38 ± 10.39 | 7703 ± 31237 |
| Threshold 200m Baseline 1 day | 7.64 ± 10.05 | 7706 ± 31235 |

**Table S5**. Fledging and independence parameters for GPS-tracked juvenile Great Cormorants (*Ph. c. sinensis*) at Lake Balaton, Hungary (2021–2023). [a] Three individuals were excluded from analyses due to lack of sufficient data.

| Individual ID | Baseline start | Baseline fixes (n) | Days to first fledging | Total fledging events (n) | Fledging distance (m) | Post-fledging care (days) | Independent days (n) | Total GPS fixes (n) |
|---|---|---|---|---|---|---|---|---|
| SP02217[a] | 19.05.22 | 72 | 3.12 | 88 | 212 | 26 | 3 | 792 |
| SP02219 | 24.05.22 | 67 | 9.37 | 6825 | 652 | 46 | 49 | 8113 |
| SP02220 | 24.05.22 | 66 | 1.54 | 7288 | 130 | 59 | 69 | 11771 |
| SP02222 | 09.05.21 | 136 | 3.42 | 18879 | 186 | 9 | 168 | 22460 |
| SP02223 | 09.05.21 | 115 | 0.27 | 12881 | 366 | 23 | 133 | 16828 |
| SP02224 | 09.05.21 | 131 | 7.35 | 1610 | 529 | 13 | 35 | 5896 |
| SP02225 | 09.05.21 | 123 | 0.94 | 18800 | 461 | 22 | 220 | 21812 |
| SP02226 | 09.05.21 | 140 | 0.17 | 45186 | 562 | 30 | 380 | 48120 |
| SP02227 | 09.05.21 | 138 | 0.44 | 21235 | 539 | 30 | 207 | 25578 |
| SP02228 | 09.05.21 | 141 | 0.92 | 8626 | 654 | 30 | 71 | 12601 |
| SP02229 | 09.05.21 | 152 | 10.75 | 3129 | 133 | 15 | 37 | 6866 |
| SP02230 | 09.05.21 | 149 | 0.31 | 17611 | 429 | 30 | 138 | 21405 |
| SP02242[a] | 24.05.22 | 66 | 3.37 | 186 | 143 | 17 | 31 | 1241 |
| SP02245 | 24.05.22 | 358 | 1.35 | 31096 | 107 | 21 | 420 | 38859 |
| SP02248 | 03.06.22 | 69 | 5.25 | 17678 | 157 | 37 | 135 | 18671 |
| SP02250[a] | 08.06.22 | 341 | 1.33 | 752 | 158 | 20 | 7 | 1215 |
| SP02252 | 03.06.22 | 415 | 0.43 | 13520 | 194 | 29 | 107 | 19148 |
| SP02253 | 17.06.22 | 242 | 3.58 | 1750 | 145 | 15 | 25 | 6077 |
| SP02255 | 24.05.23 | 245 | 3.32 | 9718 | 190 | 44 | 201 | 14521 |

Baseline starts are in DD/MM/YY format; baseline fixes are the number of GPS fixes recorded during the first 3 days post-tagging, used to establish the nest centroid. Days to first fledging is the elapsed time from tagging to the first recorded fledging event (two consecutive fixes >100 m from nest centroid). Fledging distance is the distance (m) of that first event from the nest centroid. Post-fledging care period (days) is the interval between first fledging and onset of independence (first of two consecutive days with ≥5 fledging events). Independent days and total GPS fixes refer to the tracking period following independence. Total fledging events counts all excursions >100 m from the nest centroid throughout the entire tracking period.

**Table S6.** Sensitivity analysis of the individual-optimised foraging hotspot selection algorithm to key parameter choices.

***(a) Sensitivity to target cell number***

| Target cells (n) | Core coverage (%) | Core + buffer coverage (%) | Individuals represented (%) |
|---|---|---|---|
| 200 | 54.4 | 61.8 | 100 |
| 300 | 61.8 | 70.2 | 100 |
| 400 | 65.9 | 74.8 | 100 |
| 500 | 68.2 | 77.4 | 100 |

***(b) Sensitivity to minimum GPS fix threshold per cell***

| Min. fixes per cell | Candidate cells (n) | Hotspot cells (n) | Single-individual cells (%) |
|---|---|---|---|
| 3 | 976 | 300 | 24.3 |
| 5 | 644 | 300 | 25.3 |
| 10 | 348 | 300 | 37.7 |

***(c) Sensitivity to composite score weighting (intensity : diversity)***

| Weighting (intensity : diversity) | Stage 2 coverage (%)[1] | Mean individuals per hotspot cell |
|---|---|---|
| 60 : 40 | 56.0 | 2.45 |
| 70 : 30 | 58.7 | 2.40 |
| 80 : 20 | 60.7 | 2.33 |

[1] Stage 2 coverage: proportion of all foraging GPS fixes captured by Stage 2 (composite-score-selected) cells only, excluding the mandatory Stage 1 individual-top cells.

(a) Effect of target cell number on GPS fix coverage. Core coverage represents the proportion of all foraging GPS fixes captured within selected hotspot cells; buffer coverage additionally includes the immediate surrounding cells of each hotspot and was estimated by applying the core-to-buffer ratio observed under the default parametrization (core 62.0%, core + buffer 72.0%).
(b) Effect of minimum GPS fix threshold per grid cell on the size of the candidate pool and the proportion of single-individual hotspot cells (i.e. cells visited by only one tracked individual).
(c) Effect of composite score weighting (intensity : diversity ratio) on Stage 2 coverage (proportion of all foraging fixes captured by Stage 2 cells only, excluding the mandatory Stage 1 individual-top cells) and the mean number of individuals per hotspot cell. Note that Stage 2 coverage values are therefore not directly comparable to the total coverage figures in panel (a). Default parameters (300 cells, ≥5 fixes per cell, 70:30 intensity:diversity weighting) are shown in bold. All configurations maintained 100% individual representation (at least one hotspot cell per tracked individual).

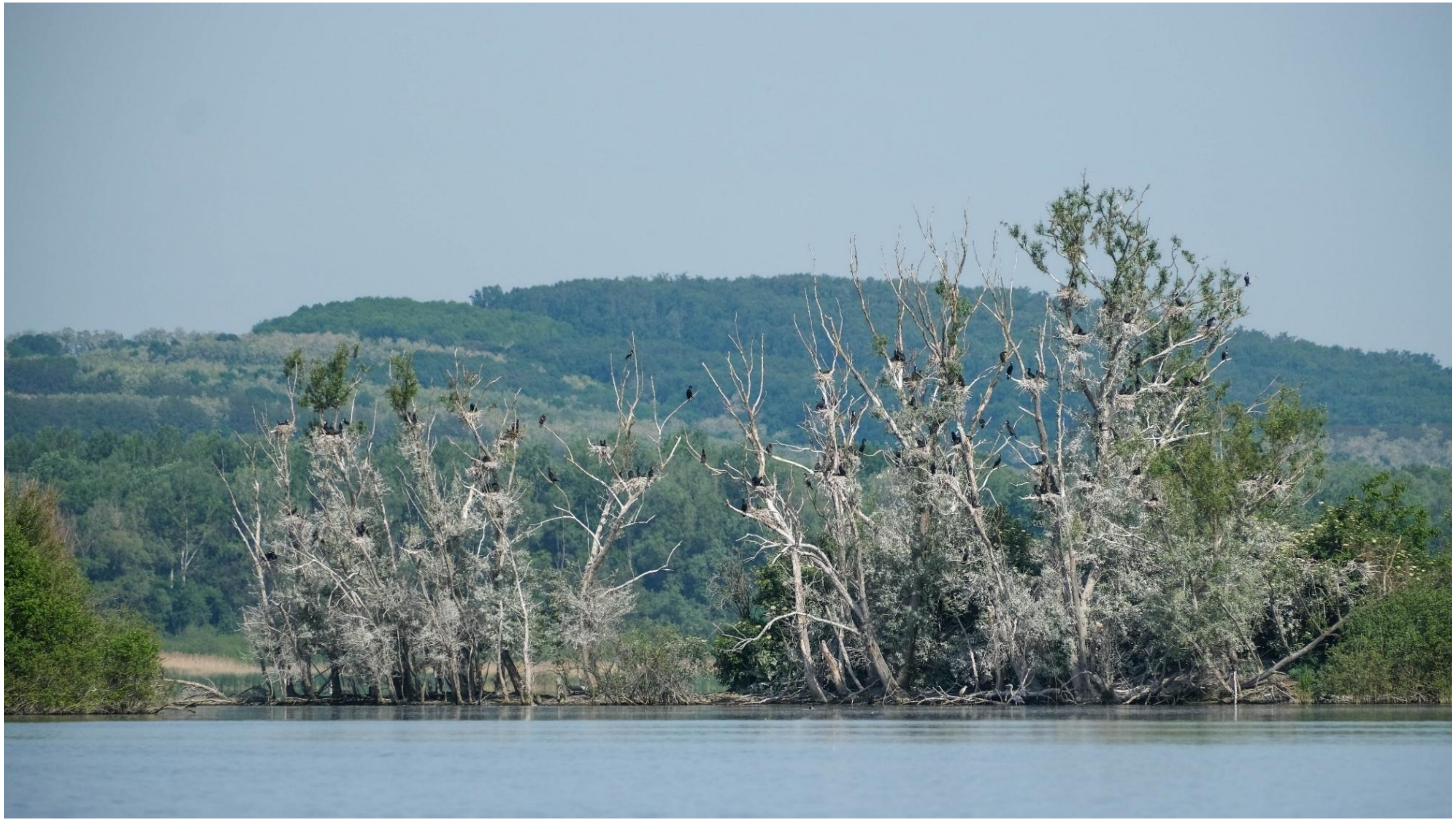

**Fig. S1.** Island tree-nesting Great Cormorants (*Ph. c. sinensis)* at the Kis-Balaton I colony

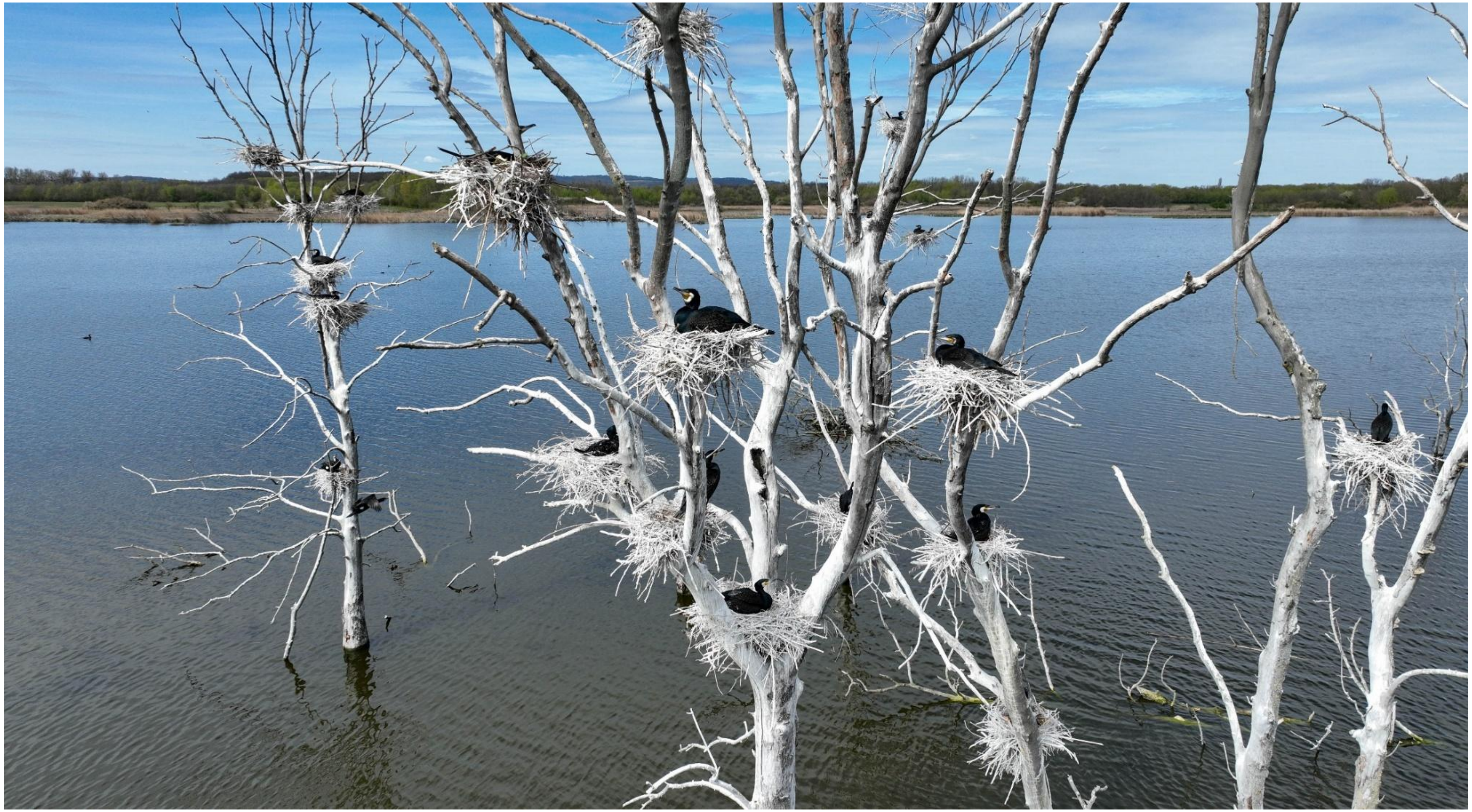

**Fig. S2.** Great Cormorants (*Ph. c. sinensis)* nesting on trees standing in water at the Kis-Balaton II colony

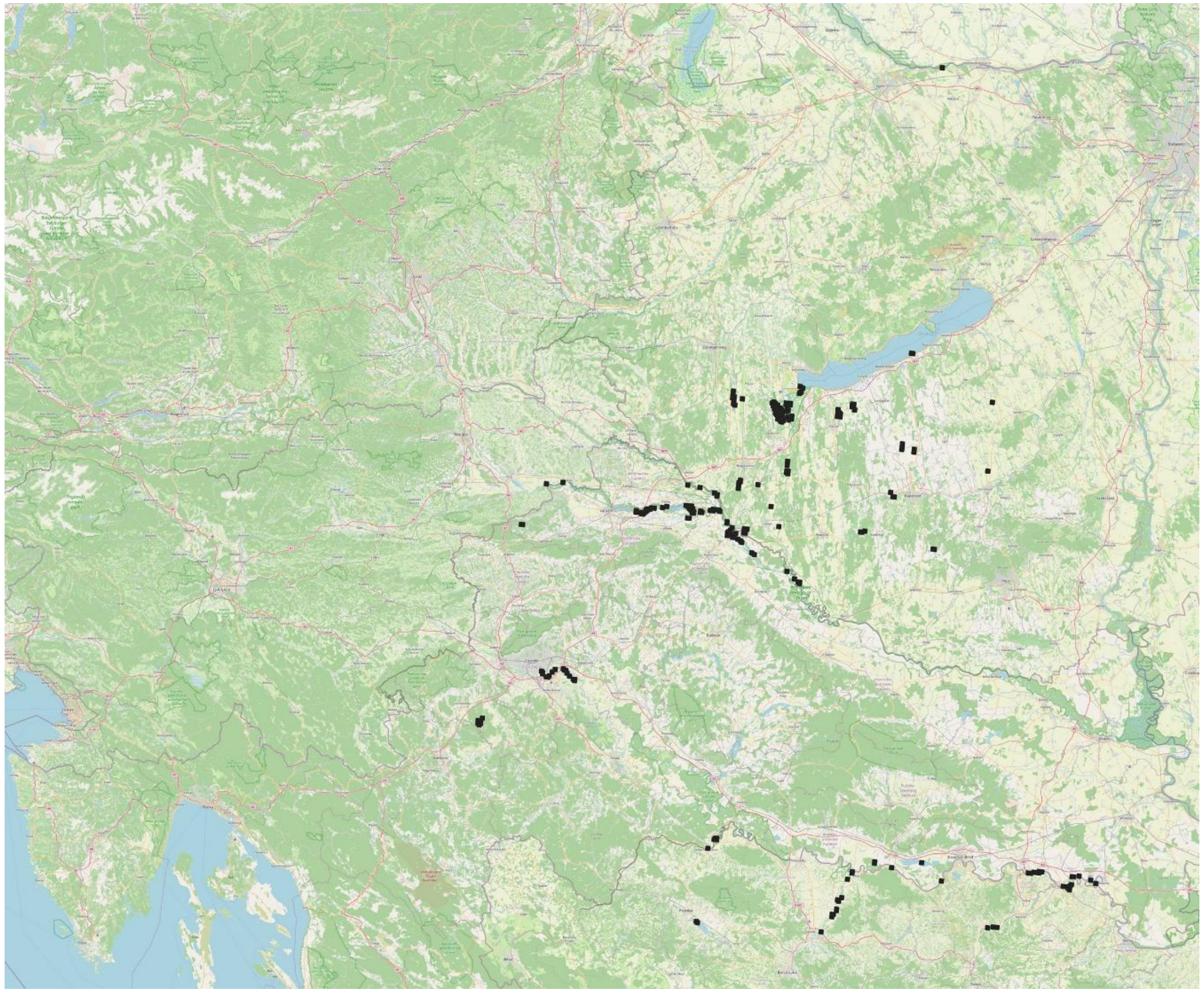

**Fig. S3.** Geographic distribution of foraging hotspot cells identified for GPS-tracked Great Cormorants (*Ph. c. sinensis*) from the Lake Balaton breeding population. Black squares indicate 500 × 500 m foraging hotspot cells, comprising core hotspot cells (*n* = 293) and surrounding buffer cells (*n* = 1,125). Basemap: OpenStreetMap contributors.
